# Acute myeloid leukemia with *NPM1*, *IDH2*, and *SETD2* mutations mimicking acute promyelocytic leukemia: A case report and literature review

**DOI:** 10.1097/MD.0000000000040222

**Published:** 2024-10-18

**Authors:** Xiang-Lei Chen, Shan-Shan Zeng

**Affiliations:** a Department of Hematology, Weifang Yidu Central Hospital, Qingzhou, China.

**Keywords:** acute myeloid leukemia, APL-like, *IDH2* mutation, *NPM1* mutation, *SETD2* mutation

## Abstract

**Rationale:**

Acute myeloid leukemia with *NPM1, IDH2*, and *SETD2* mutations can mimic acute promyelocytic leukemia (APL) and poses a challenge for the early and accurate differentiation and diagnosis of APL with *PML::RARA*.

**Patient concerns:**

A 70-year-old man was diagnosed with acute myeloid leukemia with *NPM1, IDH2*, and *SETD2* mutations.

**Diagnosis:**

APL-like acute myeloid leukemia with *NPM1*, *IDH2*, and *SETD2* mutations was made.

**Interventions:**

The patient received all-trans retinoic acid 20 mg 3 times a day for 22 days, azacitidine 100 mg subcutaneously once daily for 7 days, and venetoclax 100 mg once daily for 12 days.

**Outcomes:**

Due to economical constraints, the patient stopped further treatment, and outcome was dismal.

**Lessons:**

The comprehensive evaluation of bone marrow morphology, immunology, cytogenetics, and molecular biology is essential for the accurate diagnosis of acute myeloid leukemia.

## 1. Introduction

Acute myeloid leukemia (AML) is a heterogeneous hematologic malignancy originating from hematopoietic stem progenitor cells, often accompanied by chromosomal abnormalities and/or gene mutations. A unique subtype within AML is acute promyelocytic leukemia (APL) with *PML::RARA*, well-known among hematologists for its distinctive morphology, clinical features, and high cure rates with all-trans-retinoic acid (ATRA) combined with arsenic trioxide induction therapy. Early diagnosis relying on both morphological and immunological assessments is crucial for rescuing patients. Morphologically, APL exhibits characteristics such as numerous granules, Auer rods, and bilobed nuclei. Immunologically, CD34 and HLA-DR are often negative. In a subset of AML cases with *NPM1* mutation, a small fraction may resemble APL morphologically and immunologically. However, as they lack the *PML::RARA* fusion gene, they are termed APL-like AML and cannot be diagnosed as APL with *PML::RARA*. We encountered a case of AML with an *NPM1* mutation, along with *IDH2* and *SETD2* mutations, which morphologically and immunologically resembled APL but lacked the *PML::RARA* fusion gene, resulting in a diagnosis of APL-like AML with an *NPM1* mutation.

## 2. Case presentation

**Figure 1.**
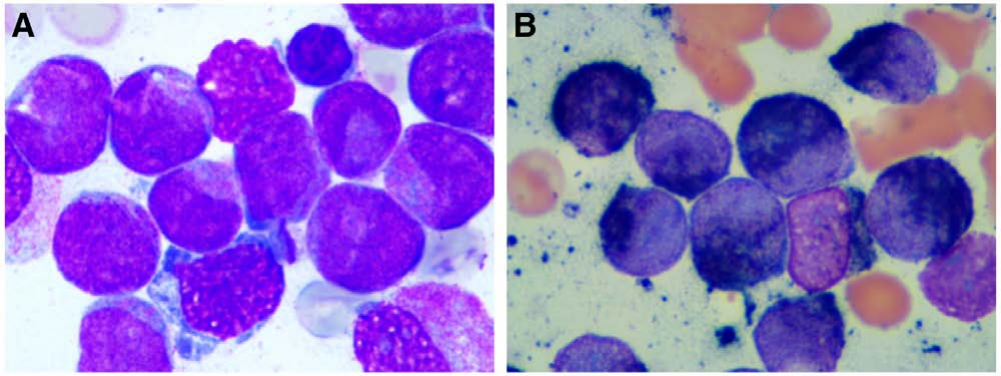
Bone marrow morphology and cytochemical staining. (A) Bone marrow shows APL-like cells. (B) APL-like cells show 100% strongly positive myeloperoxidase staining. APL = acute promyelocytic leukemia.

A 70-year-old man complaining of fever for more than 2 weeks was admitted to the Department of Hematology. On physical examination, the patient exhibits pallor. There is no tenderness over the sternum. The peripheral blood profile revealed leukocytes at 25.92 × 10^9^/L (elevated), an absolute neutrophil count of 0.18 × 10^9^/L, hemoglobin at 59 g/L (decreased), and a platelet count of 6 × 10^9^/L (decreased). The coagulation profile showed a prothrombin time of 16.1 seconds (elevated), an activated partial thromboplastin time of 30.7 seconds, a thrombin time of 14.6 seconds, and fibrinogen at 5.06 g/L (elevated). The D-dimer level was 74.18 µg/mL (elevated). The International Society on Thrombosis and Haemostasis disseminated intravascular coagulation score was 4 points. Bone marrow morphology and cytochemical staining examination (Fig. 1A) revealed marked proliferation of the granulocytic series, with a significant increase in aberrant immature granulocytic cells. These cells exhibit moderate size, abundant cytoplasm that appears pale blue, containing both coarse and fine reddish-purple granules. The nuclei are large, some irregularly shaped, demonstrating folding and invagination, with finely textured chromatin and 1 to 2 clearly visible nucleoli. Aberrant immature granulocytic cells constitute approximately 79% of the total. Myeloperoxidase staining is 100% strongly positive (Fig. 1B).

The examination for fusion genes, including *PML::RARA (L/S/V*), *NPM::RARA*, *PLZF::RARA*, *STAT5B::RARA*, *MLL::AF1P*, *MLL::AF1Q*, *MLL::AF4*, *MLL::AF6*, *MLL::AF9*, *MLL::AF10*, *MLL::AF17*, *MLL::AFX1*, *MLL::ENL*, and *MLL::ELL*, was negative. Chromosome G-banding analysis showed 46,XY [6]. Bone marrow immunophenotyping examination revealed that myeloid progenitor cells accounted for 76.23%, with decreased side scatter (SSC). These cells predominantly expressed CD33bri and cytoplasmic myeloperoxidase and partially expressed CD117, CD56, and CD64, with limited expression of CD13dim, CD123dim, and CD9. They did not express CD34, HLA-DR, CD38, CD10, CD5, CD19, CD36, CD20, CD14, CD71, CD41, CD11b, CD16, cytoplasmic CD3 (cCD3), CD11c, CD4, or CD7. Mutations in *NPM1* (p.Trp288Cysfs12), *IDH2* (p.Arg140Gln), and *SETD2* (p.Arg2024) were identified through RNA sequencing. A diagnosis of AML with *NPM1*, *IDH2*, and *SETD2* mutations mimicking APL was made.

## 3. Treatment

The treatment regimen included all-trans retinoic acid 20 mg 3 times a day for 22 days, azacitidine 100 mg subcutaneously once daily for 7 days, and venetoclax 100 mg once daily for 12 days. Due to financial constraints, the patient and their family decided to discontinue further treatment and died after 2 months.

## 4. Discussion

AML with *NPM1* mutation commonly occurs in cytogenetically normal AML and is clinically characterized by elevated white blood cell counts, increased immature cells, anemia, and thrombocytopenia. Morphologically, bone marrow often exhibits features of granulomonocytic or monocytic leukemia, while immunophenotypically, there is frequently high expression of CD33, low expression of CD13, and negativity for CD34 and HLA-DR. Genetically, *NPM1* mutations may co-occur with mutations in *FLT3*, *DNMT3A*, *IDH1*, *KRAS*, *NRA*. In terms of prognosis, younger age, normal karyotype, and absence of *FLT3-ITD* mutations are associated with better outcomes, with favorable responses to chemotherapy. However, a small subset of AML patients with *NPM1* mutation^[1]^ exhibits morphological, immunophenotypic, and clinical features resembling APL with *PML::RARA*. This similarity poses a challenge in rapidly and accurately differentiating between these 2 diseases.

The bone marrow morphology of APL-like AML with *NPM1* mutation may exhibit bilobed nuclei, prominent cytoplasmic granules, Auer rods, and occasionally, cup-like cells. Immunophenotypically, there is low or absent expression of CD34 and HLA-DR, strong positivity for CD33 and MPO, and non-expression of CD11b. Genetically, APL-like AML with *NPM1* mutation is more prone to co-occurring *TET2* or *IDH1*/*IDH2* mutations. Clinically, these cases also pose a higher risk of disseminated intravascular coagulation and early vascular events. In comparison to APL with *PML::RARA*, APL-like AML with *NPM1* mutation exhibits leukemia cells with lower SSC, frequent expression of CD4, and higher white blood cell counts. Conversely, APL with *PML::RARA* often presents with higher SSC, less expression of CD4, and more frequent overall blood cell reduction, contributing to some extent to the differentiation between the 2 leukemias. In terms of genetics, AML patients with *NPM1* mutation and co-occurring *TET2* or *IDH1*/*IDH2* mutations are more likely to display an APL-like immunophenotype. However, the manifestation of an APL-like phenotype is less probable if concurrent mutations in genes such as *DNMT3A* and *WT1* are present.^[2]^

APL with *PML::RARA* fusion gene and AML with *NPM1* mutation represent 2 distinct acute leukemias characterized by recurrent genetic abnormalities. The *PML::RARA* fusion gene exerts dominant-negative effects on target genes, leading to cellular differentiation blockade, aberrations in apoptosis and autophagy, culminating in the development of APL. Conversely, *NPM1* gene mutations result in genomic instability, loss of tumor suppressor function, inhibition of normal cell apoptosis, upregulation of MYC protein expression, abnormal expression of *HOX* genes, ultimately leading to the occurrence of acute leukemia. It is generally considered that *NPM1* gene mutations are mutually exclusive, not co-occurring with other recurrent genetic abnormalities such as the *PML::RARA* fusion gene. *PML::RARA* fusion gene may be associated with mutations in *FLT3*, *WT1*, and *NRAS* genes but is not accompanied by *NPM1* mutations. However, a study based on the Indian population^[3]^ found that up to 45% of APL patients had *NPM1* mutations, with 25% of patients exhibiting APL with isolated *NPM1* mutations, including A-type and D-type mutations. Additionally, 20% of patients had *NPM1*/*FLT3* mutations, all with A-type *NPM1* mutations. Whether these findings in the Indian population contribute to understanding the mechanisms of APL-like presentations in *NPM1*-mutated patients or if there are biases in the study, such as contamination of *NPM1*-mutated samples or differences in *NPM1* mutation detection techniques^[4]^ remains unclear. While *NPM1* is implicated in retinoic acid–induced leukemia cell differentiation,^[5]^ it is uncertain whether mutated *NPM1* retains this functionality. Whether a sole *NPM1* mutation can impede cell differentiation at the promyelocytic stage is not clear. Based on current literature, it is plausible that additional gene mutations or mechanisms are needed to hinder cell differentiation at the promyelocytic stage. Our patient, aside from the *NPM1* mutation, concurrently harbors *IDH2* and *SETD2* gene mutations.

Our patient presented with a 2-week history of pyrexia, and initial assessments, including complete blood count, coagulation parameters, bone marrow morphology, and immunophenotyping, strongly suggested a diagnosis of APL with *PML::RARA*. However, chromosomal G-banding and fusion gene screening did not support the diagnosis of APL with *PML::RARA*. Ultimately, RNA-seq testing confirmed the diagnosis of AML with *NPM1* mutation. Immunophenotypic examination in this patient revealed reduced SSC and minimal expression of CD9, characteristics more supportive of AML with *NPM1* mutation. The negative expression of CD38 was less supportive of APL with *PML::RARA*.^[6]^ Literature reports indicate that in *NPM1*-mutated acute leukemia cell lines, ATRA combined with arsenic trioxide can induce degradation of NPM1-mutated protein and apoptosis.^[7,8]^ In OCI-AML3 cell lines with *NPM1* mutation and a leukemia mouse model established from them, siRNA-mediated knockdown of mutant *NPM1* expression reduced the proportion of S-phase cells, induced leukemia cell differentiation, and increased sensitivity to ATRA and cytarabine treatment.^[8]^ Forghieri et al^[9]^ reported 3 elderly patients with *NPM1* mutation without *FLT3* or *IDH1-R132* mutations who achieved complete morphological remission with ATRA combined with low-dose cytarabine therapy. Venetoclax combined with low-dose cytarabine or azacitidine also achieved a higher molecular response rate in leukemia patients with or without *IDH2-R140*, *FLT3-ITD*, or *DNMT3A* mutations.^[10]^ *IDH1*/*2*-mutated patients treated with venetoclax combined with azacitidine had a high response rate, prolonged remission, and significantly improved survival.^[11]^ In our patient, early suspicion of APL with *PML::RARA* based on morphology and immunophenotyping prompted immediate initiation of ATRA therapy for a total of 22 days. Simultaneously, azacitidine and venetoclax were administered, but there was no improvement observed in routine blood counts and coagulation parameters. Due to the high cost, the patient opted to discontinue further treatment, preventing further observation of treatment outcomes. *SETD2* gene mutations are commonly associated with chemotherapy resistance in AML,^[12]^ the poor treatment response may be related to the *SETD2* gene mutation.

In addition to cases of AML with *NPM1* mutation, reports exist on AML with *WT1* mutation,^[13]^ AML treated with granulocyte colony-stimulating factor to induce leukocytosis,^[14]^ AML with *FLT3* mutation,^[15]^ AML with t(11;12)(p15;q13)[16], AML with inv(11)(p15;q22), AML with *MLL* rearrangement,^[16]^ and AML with MDS-like changes accompanied by 3q chromosomal deletion,^[17]^ all exhibiting morphological and immunophenotypic similarities to APL with *PML::RARA*. The 2017 “WHO Classification of Tumors of Hematopoietic and Lymphoid Tissues” introduced the APL with RAR variant subtype, encompassing genes from all 3 *RAR* family members (*RARA*, *RARB*, *RAR*G), capable of mimicking the morphology, immunophenotype, and clinical presentation of APLs other than those with *PML::RARA* positivity.

## 5. Conclusion

APL-like AML exhibits significant heterogeneity. Despite striking morphological, immunophenotypic, and coagulation parameter similarities to APL with *PML::RARA*, the absence of the *PML::RARA* fusion gene necessitates the exclusion of a diagnosis of APL with *PML::RARA*. Clinical practitioners should fully appreciate the significance of a comprehensive diagnosis involving morphology, immunophenotyping, cytogenetics, and molecular testing, facilitating effective communication with patients. Given that these cases are often reported as individual cases or case series, collaboration among large medical centers, diagnostic facilities, and primary care institutions is essential. Establishing efficient communication and information-sharing mechanisms, along with convenient specimen preservation protocols, is crucial for studying the unique features and pathogenesis of these cases. This collaborative effort aims to develop rapid and accurate diagnostic methods, ultimately contributing to the refinement of treatment strategies.^[6]^

## Author contributions

**Conceptualization:** Xiang-Lei Chen, Shan-Shan Zeng.

**Data curation:** Xiang-Lei Chen.

**Formal analysis:** Xiang-Lei Chen.

**Writing – original draft:** Xiang-Lei Chen.

**Writing – review & editing:** Xiang-Lei Chen, Shan-Shan Zeng.
